# Immediate and Longer-Term Changes in the Mental Health and Well-being of Older Adults in England During the COVID-19 Pandemic

**DOI:** 10.1001/jamapsychiatry.2021.3749

**Published:** 2021-12-22

**Authors:** Paola Zaninotto, Eleonora Iob, Panayotes Demakakos, Andrew Steptoe

**Affiliations:** 1 Department of Epidemiology and Public Health, University College London, London, United Kingdom; 2 Department of Behavioural Science and Health, University College London, London, United Kingdom

## Abstract

**Importance:**

Despite the emphasis placed on the psychological impact of the COVID-19 pandemic, evidence from representative studies of older adults including pre–COVID-19 data and repeated assessments during the pandemic is scarce.

**Objective:**

To examine changes in mental health and well-being before and during the initial and later phases of the COVID-19 pandemic and test whether patterns varied with sociodemographic characteristics in a representative sample of older adults living in England.

**Design, Setting, and Participants:**

This longitudinal cohort study analyzed data from 5146 older adults participating in the English Longitudinal Study of Ageing who provided data before the COVID-19 pandemic (2018 and 2019) and at 2 occasions in 2020 (June or July as well as November or December).

**Exposures:**

The COVID-19 pandemic and sociodemographic characteristics, including sex, age, partnership status, and socioeconomic position.

**Main Outcomes and Measures:**

Changes in depression (8-item Centre for Epidemiological Studies Depression scale), anxiety (7-item Generalized Anxiety Disorder scale), quality of life (12-item Control, Autonomy, Self-realization, and Pleasure scale), and loneliness (3-item Revised University of California, Los Angeles, loneliness scale) were tested before and during the COVID-19 pandemic using fixed-effects regression models.

**Results:**

Of 5146 included participants, 2723 (52.9%) were women, 4773 (92.8%) were White, and the mean (SD) age was 67.7 (10.6) years. The prevalence of clinically significant depressive symptoms increased from 12.5% (95% CI, 11.5-13.4) before the COVID-19 pandemic to 22.6% (95% CI, 21.6-23.6) in June and July 2020, with a further rise to 28.5% (95% CI, 27.6-29.5) in November and December 2020. This was accompanied by increased loneliness and deterioration in quality of life. The prevalence of anxiety rose from 9.4% (95% CI, 8.8-9.9) to 10.9% (95% CI, 10.3-11.5) from June and July 2020 to November and December 2020. Women and nonpartnered people experienced worse changes in mental health. Participants with less wealth had the lowest levels of mental health before and during the COVID-19 pandemic. Higher socioeconomic groups had better mental health overall but responded to the COVID-19 pandemic with more negative changes.

**Conclusions and Relevance:**

In this longitudinal cohort study of older adults living in England, mental health and well-being continued to worsen as the COVID-19 pandemic progressed, and socioeconomic inequalities persisted. Women and nonpartnered people experienced greater deterioration in mental health.

## Key Points

**Question:**

How have the mental health and well-being of older adults in England changed during the COVID-19 pandemic compared with prepandemic levels?

**Findings:**

This cohort study including 5146 older adults participating in the English Longitudinal Study of Ageing found that levels of depression, loneliness, and poor quality of life increased significantly during June and July 2020 compared with prepandemic levels and continued to deteriorate during the second national lockdown in November and December 2020, with further increases in anxiety symptoms from June and July 2020 to November and December 2020. Inequalities in experiences of mental ill health during the COVID-19 pandemic were evident, with women, individuals living alone, and those with less wealth being particularly vulnerable.

**Meaning:**

Older individuals did not adapt well to the new psychosocial stressors introduced by the pandemic; policies should be in place for the immediate provision of targeted psychological interventions to support older people, and access to digital mental health services should be improved.

## Teaser

This cohort study examines changes in mental health and well-being before and during the initial and later phases of the COVID-19 pandemic and tests whether patterns varied with sociodemographic characteristics in a representative sample of older adults living in England.

## Introduction

It is now well documented that the COVID-19 pandemic has significantly affected the mental health and well-being of the adult population all around the world.^1,2^ Stress directly related to the disease and worries about disruption to health care services, employment, financial security, and limitations to social contacts have contributed to psychological distress, as reported by internet studies initiated after the onset of the COVID-19 lockdown.^3,4,5,6^ Such studies lack information about mental health and well-being before the COVID-19 pandemic, so increases in distress are inferred rather than measured directly. Data collection online rules out participants who do not have internet access, including socially marginalized groups and sectors of the older population.^7^ Nevertheless, these findings have been corroborated by longitudinal studies that compare experiences during the early months of the pandemic with data collected in the past years.^8^ Consistent with studies in other countries,^9,10^ analyses of the UK Longitudinal Household Study confirm increases in psychological distress during the early months of the COVID-19 pandemic in the UK.^8,11^ These results have also been corroborated by individual-specific counterfactual predictions accounting for pre-existing trends in mental health.^12^

Older adults are at increased risk of serious illness and death following COVID-19 infection^13^ and are vulnerable to social isolation and loss of access to social and health care. These experiences may lead to poor mental health and well-being that are in turn associated with cognitive decline,^14^ incident dementia,^15^ mortality,^16^ and several physical health conditions.^17,18^ However, some studies have reported little deterioration in mental health and well-being among older people in the UK,^8,11,19^ US, Netherlands, and Sweden.^19,20,21^ but levels of loneliness were higher than before the COVID-19 pandemic.^20^ Notably, most studies to date, including those discussed above, have been conducted in the early months of the COVID-19 pandemic, but patterns of mental health and well-being may change with repeated social distancing regulations, as have occurred in the UK in 2020. Prolonged restrictions and persistence in infection rates may have taxed older people’s capacity to adapt, resulting in increasing levels of mental distress. In the UK, overall levels of psychological distress began to recover in July through to October 2020 but not to pre–COVID-19 pandemic levels.^22^ However, the impact of the second national lockdown that came into force in England in November 2020 and subsequent waves of COVID-19 remains unclear. More detailed assessments of mental health and well-being among older adults throughout the COVID-19 pandemic are needed to establish whether older people are indeed more resilient to the psychological impact of the COVID-19 crisis.

Using data from the English Longitudinal Study of Ageing (ELSA) COVID-19 substudy carried out in June and July 2020 and in November and December 2020 (online and by telephone), we evaluated whether mental health (depressive symptoms and anxiety) and well-being (quality of life and loneliness) were affected at 2 time points during the COVID-19 pandemic compared with previous years. We also investigated whether patterns varied with age, sex, socioeconomic status, and partnership status, as found in surveys that have involved adults throughout the age spectrum.

## Methods

### Sample

The data came from ELSA, an ongoing representative study of older adults 50 years and older living in England^23,24^ interviewed every 2 years since 2002 (9 waves of data have been collected). In addition, the ELSA COVID-19 substudy collected data in June and July 2020 and in November and December 2020.^25^ The response rate was high in both waves (cross-sectional, 75%; longitudinal, 94%). For the purpose of the present study, we created a longitudinal sample including 5146 respondents of the ELSA COVID-19 substudy (52 years and older in 2020) who participated in both COVID-19 survey periods and in the most recent regular ELSA wave previous to COVID-19 (wave 9 in 2018 and 2019). Vaccination began early in 2021 in the United Kingdom, and therefore, none of the participants had been vaccinated when the data were collected in 2020. Compared with the full ELSA sample at wave 9, the participants included in the analytical sample were slightly older and more likely to be women, retired, and identify as White individuals, and they had higher wealth and better self-rated health (eTable 1 in the Supplement). Therefore, all analyses were weighted using the longitudinal survey weights to account for nonresponse to the ELSA COVID-19 substudy survey and match the latest population estimates for age, sex, race and ethnicity, and region in England.^25^ All respondents provided informed consent—written for those who completed the survey online and oral for those who completed the survey by telephone. Ethical approval for the regular ELSA study was obtained from the National Research Ethics Service. The ELSA COVID-19 substudy was approved by the University College London Research Ethics Committee. The study followed the Strengthening the Reporting of Observational Studies in Epidemiology (STROBE) reporting guideline.

### Measures

#### Outcomes

Depressive symptoms were ascertained using the 8-item Centre for Epidemiological Studies Depression (CESD-8) scale. We created a binary variable using a cutoff point of 4 or more symptoms to identify likely cases of clinical depression, which is equivalent to the conventional threshold of 16 or higher on the full 20-item CESD scale.^26^ Quality of life was measured using the 12-item version of the Control, Autonomy, Self-realization, and Pleasure (CASP) scale. The resulting item scores were summed to create an index of quality of life (range, 1-48), where higher scores indicate poorer well-being. Loneliness was assessed using the 3-item revised University of California, Los Angeles, loneliness scale and an additional item asking participants how often they feel lonely. The individual item scores were summed together to produce a total score (range, 1-12), with higher values indicating greater loneliness. Anxiety was measured using the 7-item Generalized Anxiety Disorder (GAD-7) scale. We used a total score of 10 or greater as a cutoff point for identifying cases of generalized anxiety disorder. Further details about the mental health scales are provided in the eMethods in the Supplement.

#### Sociodemographic Characteristics

Sociodemographic characteristics considered in the analysis were age (50 to 59 years, 60 to 74 years, and 75 years and older), sex, wealth tertiles (first tertile = lowest wealth; third tertile = highest wealth), and whether the participant had a partner. Further details about the measurement and coding of these data and other variables used in the descriptive analyses are provided in the eMethods in the Supplement.

### Statistical Analysis

We examined changes within an individual’s mental health during the COVID-19 pandemic using 2-way fixed-effects regression models. A linear model was used to estimate changes in the total scores of quality of life and loneliness as well as in the probability of the binary depression and anxiety scores. For the binary outcomes, a linear probability model was chosen over a logistic fixed-effects model, since this approach would exclude those who had concordant scores across all waves, thereby affecting the representativeness and statistical power of the analysis. Linear probability models with fixed effects have been shown to produce accurate estimates and predicted probabilities, particularly when the prevalence of the outcome in the sample is less than 30%,^27^ as in the case of our study (eTable 2 in the Supplement). First, we estimated changes in depression, quality of life, and loneliness during the COVID-19 pandemic using 2 binary independent variables indicating whether the outcome was measured at the first or second COVID-19 wave (vs before the COVID-19 pandemic). For anxiety, we used a binary variable indicating whether the outcome was measured at the first vs second COVID-19 wave, as this scale was not included previously in the regular ELSA survey. We then repeated this analysis using the standardized outcome scores to enable direct comparisons across the outcomes. Second, we tested interaction effects between a COVID-19 period indicator for whether the outcome was measured before or during the COVID-19 pandemic (survey periods 1 or 2) and the 4 sociodemographic factors described above to understand whether and how the mean change in each mental health outcome before and during the COVID-19 pandemic might vary across different sociodemographic groups. For each outcome, we first tested each interaction effect individually and then fitted a mutually adjusted model including all interactions between the COVID-19 period indicator and the sociodemographic factors (eMethods in the Supplement). For the significant interaction effects found in the mutually adjusted models, we then produced graphs of the predicted outcome values by the selected sociodemographic groups. Missing data on all variables were estimated using multiple imputation by chained equations (eMethods in the Supplement). Several sensitivity analyses were tested, as detailed in the eMethods in the Supplement. Data management and regression analyses were conducted in Stata version 16 (StataCorp). Graphical and multiple imputation by chained equations analyses were performed in R version 4.0.2 (The R Foundation).

## Results

### Sample Characteristics

The characteristics of the analytical sample are reported in the Table. Of 5146 included participants, 2723 (52.9%) were women, 4773 (92.8%) were White, and the mean (SD) age was 67.7 (10.6) years. Descriptive statistics of the mental health outcomes across the assessments are reported in eTable 2 in the Supplement.

**Table.** Characteristics of the English Longitudinal Study of Ageing COVID-19 Substudy Sample^a^

Characteristic	No. (%)
Total, No.	5146
Sex	
Men	2423 (47.1)
Women	2723 (52.9)
Age group, y	
52-59	1640 (31.9)
60-74	2282 (44.3)
≥75	1224 (23.8)
Race and ethnicity^b^	
Asian, Black, and minority ethnic	373 (7.2)
Not Asian, Black, or minority ethnic^c^	4773 (92.8)
Partnership	
Partnered	3881 (75.4)
Nonpartnered	1265 (24.6)
Education	
≥College degree	1055 (20.5)
Completed compulsory school/some college	2401 (46.7)
<Compulsory school	1690 (32.8)
Employment status	
Employed	2006 (39.0)
Retired	2646 (51.4)
Not working^d^	494 (9.6)
Wealth (tertiles)	
First (poorest)	2169 (42.6)
Second	1524 (29.9)
Third (richest)	1402 (27.5)
Home tenure	
Owns outright	3078 (59.9)
Owns with mortgage	1125 (21.9)
Rents	937 (18.2)
Limiting long-standing illness	
No	2490 (48.4)
Yes	2656 (51.6)
Confirmed or suspected COVID-19 infection^e^	
No	4867 (94.6)
Yes	279 (5.4)
Elevated depressive symptoms (CESD-8 score ≥4)^f^	
No	3985 (77.4)
Yes	1161 (22.6)
Poor quality of life (CASP-12 score), mean (SD; range)^f^^,^^g^	22.53 (6.53; 12-47)
Loneliness, mean (SD; range)^f^^,^^h^	5.65 (2.07; 2-12)
Anxiety (GAD-7 score ≥10)^f^	
No	4661 (90.6)
Yes	482 (9.4)

Abbreviations: CASP-12, 12-item Control, Autonomy, Self-realization, and Pleasure scale; CESD-8, 8-item Centre for Epidemiological Studies Depression scale; GAD-7, 7-item Generalized Anxiety Disorder scale.

^a^ Results based on 20 imputed data sets; percentages and means are estimated using sampling weights. Only percentages are provided for categorical variables, as counts vary across the imputed data sets.

^b^ Race and ethnicity data were collected via self-report as Asian, Black, and minority ethnic or not Asian, Black, and minority ethnic.

^c^ The percentage of people who were not Asian, Black, or minority ethnic in the sample is in line with that found in other representative samples of the UK population.^11^

^d^ Permanently unable to work, looking after home and family, or currently out of work.

^e^ Prevalence of COVID-19 infections across both the COVID-19 study periods (June and July 2020 as well as November and December 2020).

^f^ Mental health scores at the first COVID-19 study period (June and July 2020).

^g^ Higher scores indicate poorer quality of life.

^h^ Measured on the UCLA Loneliness scale.

### Mental Health Outcomes Before and During COVID-19

**Figure 1.**
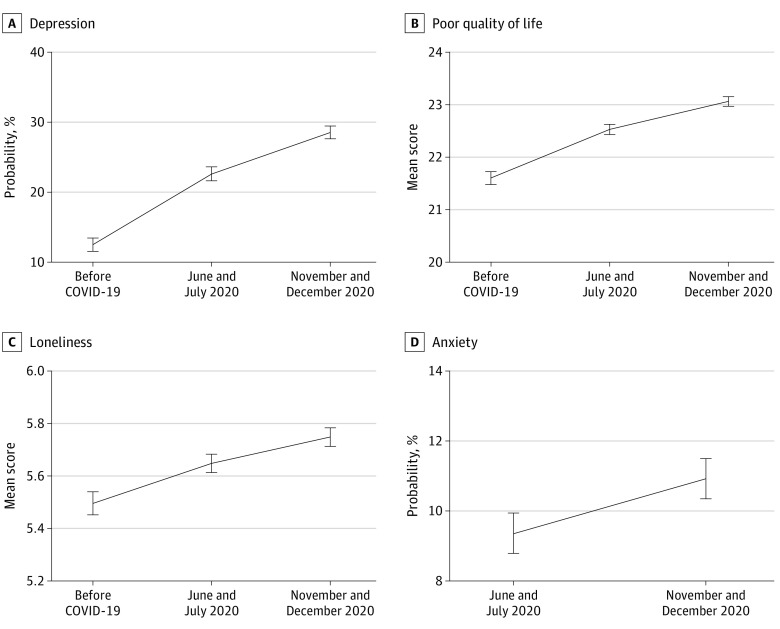
Predicted Outcome Trajectories Before and During the COVID-19 Pandemic A total of 5146 individuals participated in the English Longitudinal Study of Ageing COVID-19 longitudinal sample. Weighted pooled estimates are from 2-way fixed-effects linear models across 20 imputed data sets. Error bars indicate 95% CIs. A, Depression scores were calculated using the 8-item Center for Epidemiologic Studies Depression Scale (CESD-8) (binary score defined as total CESD-8 score greater than or equal to 4). B, Quality of life scores were calculated using the 12-item Control, Autonomy, Self-realization and Pleasure scale (total continuous score). C, Loneliness scores were calculated using the University of California, Los Angeles, loneliness scale plus additional loneliness question (total continuous score). D, Anxiety scores were calculated using the 7-item Generalized Anxiety Disorder Assessment (GAD-7) (binary score defined as total GAD-7 score equal to or greater than 10).

The predicted trajectories of the mental health outcomes before (ie, wave 9) and during the COVID-19 pandemic are reported in Figure 1 and in eTable 3 and eFigure 1 in the Supplement. Compared with pre–COVID-19 pandemic levels, all mental health outcomes deteriorated during the first COVID-19 survey period (June and July 2020) and continued to worsen through the second survey period (November and December 2020). The probability of depression increased from 12.5% (95% CI, 11.5-13.4) before the COVID-19 pandemic to 22.6% (95% CI, 21.6-23.6) in June and July 2020 and to 28.5% (95% CI, 27.6-29.5) in November and December 2020. This was accompanied by increases in the mean total scores of loneliness (before COVID-19 pandemic: 5.50; 95% CI, 5.45-5.54; June and July 2020: 5.65; 95% CI, 5.61-5.68; November and December 2020: 5.75; 95% CI, 5.71-5.78) and poor quality of life (before COVID-19 pandemic: 21.6; 95% CI, 21.48-21.72; June and July 2020: 22.5; 95% CI, 22.43-22.63; November and December 2020: 23.1; 95% CI, 22.97-23.16). Further, there was an increase in the levels of anxiety in the sample across the 2 COVID-19 survey periods from 9.4% (95% CI, 8.8-9.9) in June and July 2020 to 10.9% (95% CI, 10.3-11.5) in November and December 2020. The observed changes in mental health before and during the COVID-19 pandemic mirrored those of the fixed-effects analysis (eTable 3 in the Supplement).

### Within-Individual Changes in Mental Health

The estimated changes in mental health are reported in eTable 4 in the Supplement. During June and July 2020, the probability of depression increased by 10 percentage points (95% CI, 0.09-0.12), corresponding to an increase of 81% compared with pre–COVID-19 pandemic scores (computed as change score [ie, slope] divided by baseline value [ie, intercept] and multiplied by 100). Ratings of poor quality of life increased by 0.93 points (95% CI, 0.73-1.12), and loneliness increased by 0.15 points (95% CI, 0.08-0.22), representing an increase of 4.3% and 2.8%, respectively, compared with pre–COVID-19 pandemic scores. Significant changes in mental health were also found in November and December 2020. Compared with survey responses in June and July 2020, there was an increase of 6 percentage points (95% CI, 0.04-0.08) in the probability of depression, an increase of 0.53 points in ratings of poor quality of life (95% CI, 0.38-0.69), and an increase of 0.10 points in loneliness (95% CI, 0.04-0.16), corresponding to a change of 26.2%, 2.4%, and 1.8%, respectively. Further, the probability of anxiety increased by almost 2 percentage points (0.02; 95% CI, 0-0.03) during November and December 2020, indicating an increase of 16.6% compared with the levels of anxiety during June and July 2020. Of note, the increase in the levels of depression during COVID-19 was considerably larger than the change in the other outcomes also when considering the total number of depressive symptoms (ie, 45% increase before COVID-19 vs first COVID-19 survey period and 13% increase between COVID-19 survey periods; eTable 6 in the Supplement).

**Figure 2.**
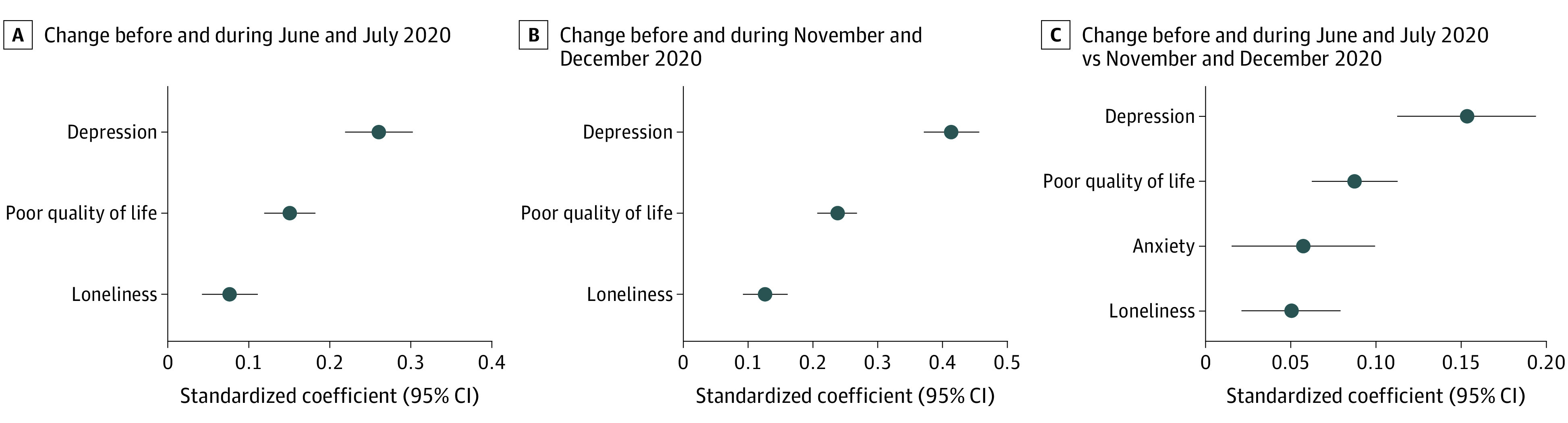
Standardized Changes in Mental Health Before and During the COVID-19 Pandemic A total of 5146 individuals participated in the English Longitudinal Study of Ageing COVID-19 longitudinal sample. Weighted pooled estimates are from 2-way fixed-effects linear models across 20 imputed data sets. Depression scores were calculated using the 8-item Center for Epidemiologic Studies Depression Scale (CESD-8) (binary score defined as total CESD-8 score greater than or equal to 4). Quality of life scores were calculated using the 12-item Control, Autonomy, Self-realization and Pleasure scale (total continuous score). Loneliness scores were calculated using the University of California, Los Angeles, loneliness scale plus additional loneliness question (total continuous score). Anxiety scores were calculated using the 7-item Generalized Anxiety Disorder Assessment (GAD-7) (binary score defined as total GAD-7 score equal to or greater than 10).

The standardized change scores of the mental health outcomes before and during the COVID-19 pandemic are illustrated in Figure 2. In line with previous results, the sharpest deterioration before and during the COVID-19 pandemic was found for depression (β = 0.26; 95% CI, 0.22-0.30), followed by poor quality of life (β = 0.15; 95% CI, 0.12-0.18) and loneliness (β = 0.08; 95% CI, 0.04-0.11). Depression also showed the largest increase across the 2 COVID-19 survey periods (β = 0.15; 95% CI, 0.11-0.19), followed by poor quality of life (β = 0.09; 95% CI, 0.06-0.11), anxiety (β = 0.06; 95% CI, 0.01-0.10), and loneliness (β = 0.05; 95% CI, 0.02-0.08).

### Changes in Mental Health and Well-being Across Different Sociodemographic Groups

**Figure 3.**
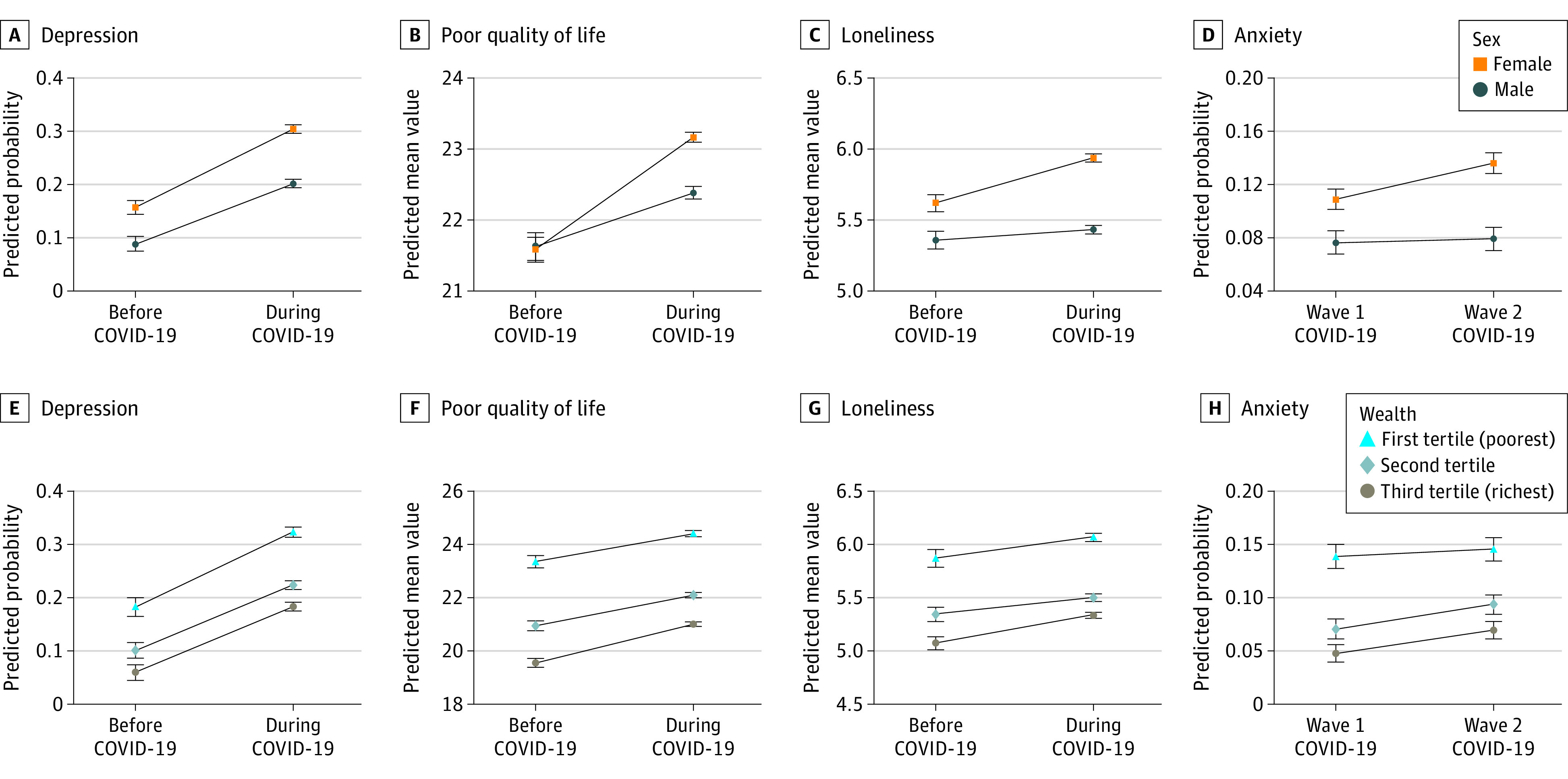
Interaction Effects Between Changes in Mental Health and Sex and Wealth A total of 5146 individuals participated in the English Longitudinal Study of Ageing COVID-19 longitudinal sample. Predicted values of the outcomes by sociodemographic characteristics were derived from mutually adjusted 2-way fixed-effects linear models; weighted pooled estimates are across 20 imputed data sets. Error bars indicate 95% CIs.

The results of interaction effects between the COVID-19 period (eTable 5 in the Supplement) and sex, wealth, partnership, and age provide evidence for some heterogeneity in the mental health impact of the pandemic (Figure 3). Women experienced worse changes in mental health than men across all outcomes. The increase in depression was 3 percentage points (0.03; 95% CI, 0-0.06) higher in women than in men. Mean ratings of poor quality of life during the COVID-19 assessments were 0.86 points (95% CI, 0.48-1.24) higher in women compared with men. The most notable sex differences were found for loneliness (interaction effect = 0.24; 95% CI, 0.11-0.36) and anxiety (interaction effect = 0.03; 95% CI, 0-0.05), which increased among women but remained almost stable in men (Figure 3). The increase in poor quality of life and loneliness during the COVID-19 pandemic was smaller for participants in the poorest wealth group compared with those in the richest wealth group (poor quality of life: interaction effect = −0.53; 95% CI, −0.95 to −0.11; loneliness: interaction effect = −0.15; 95% CI, −0.29 to 0). However, participants with lower wealth had worse mental health than those with higher wealth both before and during the COVID-19 pandemic across all outcomes considered in the analyses (Figure 3); this suggests that socioeconomic inequalities in mental health have persisted during the COVID-19 pandemic. Further, ratings of loneliness increased significantly among participants who did not have a partner (and were living alone) (interaction effect = 0.23; 95% CI, 0.07-0.39) but remained almost stable in those who had a partner (eFigure 2 in the Supplement). We did not find marked age differences. The only exception was for depression (eFigure 2 in the Supplement), which showed a smaller increase in the oldest age group (75 years and older) than in the youngest age group (aged 50 to 59 years) (*b* = −0.05; 95% CI, −0.09 to −0.01).

### Sensitivity Analyses

First, when assessing the robustness of the results of the main analyses with the imputed data and the assumptions of the fixed-effects regression models, we found that results were consistent with those of the main analyses presented above (eResults and eTables 6 to 12 in the Supplement). Second, the estimated changes in mental health during the COVID-19 pandemic (June and July 2020 as well as November and December 2020) vs before (wave 9) adjusted for earlier trends (wave 4 to 8) aligned closely with those observed in the main analysis (eResults, eTables 13 and 14, and eFigure 3 in the Supplement). Of note, the estimated prevalence of depression in the sample during COVID-19 was also considerably larger than the average depression prevalence over the preceding 11 to 12 years (average prevalence: before COVID-19 [2008-2019], 16%; during COVID-19 [2020], 26%), providing corroborative evidence of marked increases in depressive symptoms during the COVID-19 pandemic. Although changes in quality of life and loneliness during the COVID-19 pandemic were smaller than changes in depression, their magnitude was similar to the difference in loneliness and quality of life scores between people with and without chronic physical illnesses (eg, cancer, cardiovascular disease) at wave 9 (eTable 15 in the Supplement).

## Discussion

Using a nationally representative sample of older people living in private households in England, we investigated longitudinal changes in mental health before and during the initial and later phases of the COVID-19 pandemic. Our results provided clear evidence for an overall deterioration in all mental health outcomes, which persisted throughout the course of the COVID-19 pandemic in 2020. Levels of depression, poor quality of life, and loneliness increased significantly during June and July 2020 and again in November and December 2020 compared with pre–COVID-19 pandemic levels. The largest changes were observed for depression (β range, 0.26 to 0.41), followed by poor quality of life (β range, 0.15 to 0.24), and loneliness (β range, 0.08 to 0.13). We also found a significant increase in the levels of anxiety during the COVID-19 pandemic (β = 0.06; 95% CI, 0.01-0.10). Furthermore, we showed that changes in mental health varied across distinct sociodemographic groups. Deterioration of mental health was greater in women than in men across all outcomes. Participants with less wealth had lower levels of mental health than those in the highest wealth group, before and during the COVID-19 pandemic. Nevertheless, people with higher wealth experienced more negative changes in quality of life and loneliness throughout the COVID-19 pandemic. Mean ratings of loneliness increased among people who did not have a partner and were living alone.

The novelty of this study lies in the analysis of changes in mental health outcomes among older people living in the community before and during the early and later stages of the COVID-19 pandemic and in the identification of vulnerable groups therein. Studies involving repeated assessments have suggested that the highest levels of distress were experienced early in the COVID-19 pandemic, with recovery during the summer months of 2020.^22,28^ We showed that the mental health and well-being of older adults continued to worsen in the second lockdown period. Recent studies on older people have not been able to analyze large nationally representative samples with pre–COVID-19 data^29,30^ or did not include data beyond the first few months of the COVID-19 pandemic.^19,20,21^ Therefore, our results showing lower levels of mental health during the summer and again in Autumn 2020 compared with pre–COVID-19 pandemic data are novel. Of note, such changes are not in line with the trends that these measures typically display as people age. Although quality of life has been previously shown to decrease as people get older,^31^ prevalence rates of depression and anxiety tend to fall with age,^32,33^ and changes in loneliness are generally unrelated to age.^34^ Our sensitivity analyses further suggest that the increase in psychological distress during the COVID-19 pandemic observed in our study occurred against a slight downward trend in all mental health outcomes over the preceding years.

Our result regarding vulnerable groups, including women, single, widowed, and divorced people, and those with low wealth, is in line with other studies.^4,8,11,29,30^ It is worth noting that the pre–COVID-19 pandemic prevalence of depression and anxiety disorders was considerably higher in women than in men across all age groups.^32,33^ However, our longitudinal analysis demonstrated that women not only reported poorer mental health than men before and during the COVID-19 pandemic but also exhibited more negative responses throughout the pandemic. Contrary to previous research suggesting that older adults may be resilient to the mental health impact of the COVID-19 crisis,^11,19,22^ our study showed that changes in well-being and anxiety were similar across middle-aged and older adults, with only a slightly lower increase in depression found in those in the oldest age group (75 years and older) than in people in the youngest age group (aged 52 to 59 years). It is possible that the younger group continued to experience more stressors as further lockdown measures were included and did not have the time to adapt to circumstances.

To our knowledge, our finding that people from high socioeconomic groups experienced a steep decline in quality of life and increased loneliness has not been previously reported. A possible explanation might be that wealthier people have been more affected by social restrictions and the cessation of social and cultural activities than people in less affluent groups, which in turn might have resulted in impaired quality of life and greater loneliness. It has also been reported that older people with wealth held in risky assets have been severely hit by fluctuations in the stock markets.^35^ Many older individuals have private pension savings that are exposed to market risk, and findings from the ELSA COVID-19 substudy showed that 32% of people believe the value of their pension is considerably lower than before the COVID-19 crisis,^35^ which in turn can affect their overall evaluation of quality of life. However, it should be noted that despite the greater increase in mental ill health, the levels of depression, anxiety, loneliness, and poor quality of life did not reach those of less affluent groups.

### Strengths and Limitations

Our study has many strengths. It is based on a nationally representative sample of older individuals living in England, with pre–COVID-19 data. The response rate at both assessments was very high, and the data were collected through online surveys and by telephone interviews so that older adults who were unable to access the internet were not excluded. Therefore, the results are generalizable to the English population 50 years and older. Our study also has limitations. The sample was predominantly White, and therefore, the findings are not generalizable to racial and ethnic minority populations. Further, the self-reported nature of the mental health outcomes might have introduced some measurement error to the results. Another possible limitation of our study is that the first round of data collection took place as the first COVID-19 lockdown in England was easing. It is possible that mental health problems were much higher in April and May 2020, and we were not able to assess this. Nevertheless, our study shows the importance of providing resources to manage or attenuate the adverse mental health impact of the COVID-19 pandemic on older people living in the community.

## Conclusions

In this longitudinal cohort study of older adults living in England, increases in the prevalence of depression suggest that large numbers of older adults might require additional mental health support during the COVID-19 pandemic. This finding underscores the need to improve screening for mental health problems and access to psychological support for older people. Although changes in quality of life and loneliness during the COVID-19 pandemic were smaller than changes in depression, their magnitude was similar to the difference in loneliness and quality of life scores between people with and without chronic physical illnesses (eg, cancer, cardiovascular disease) at wave 9 of the ELSA study. Such changes could therefore place additional pressure on already stretched mental health services. It is known that psychological factors play an important role in adherence to public health measures (eg, vaccination) and in how people cope with the threat of infection and consequent losses^36^ as well as influencing physical health and health behaviors. Policies should be in place for the immediate provision of targeted interventions to support the mental and physical health of older people, in particular women, nonpartnered people, and those from low socioeconomic groups. Access to digital mental health services should be improved to reach older people with poorer digital resources. As the COVID-19 crisis extends beyond 2020, there is a need to sustain the mental health of older people in the population and to plan health and social support services as face-to-face contact becomes more feasible.

## Notes

Supplement. eMethods. **eResults.** Sensitivity analyses. **eTable 1.** Comparison of the characteristics of ELSA participants included in the analytical sample vs those not included at wave 9. **eTable 2.** Descriptive statistics of the mental health outcomes before and during COVID-19. **eTable 3.** Predicted values of the mental health outcomes before and during COVID-19 (fixed-effects model results). **eFigure 1.** Predicted values of the mental health outcomes before and during the COVID-19 pandemic. **eTable 4.** Two-way fixed-effects models: changes in mental health before and during the COVID-19 pandemic. **eTable 5.** Two-way fixed-effects models: interaction effects between changes in mental health and sociodemographic characteristics. **eFigure 2.** Fixed-effects models: interaction effects between changes in mental health and sociodemographic characteristics. **eTable 6.** Two-way fixed-effects models: changes in the total scores of depression and anxiety before and during the COVID-19 pandemic. **eTable 7.** Two-way fixed-effects models: interaction effects between changes in the total scores of depression and anxiety and sociodemographic characteristics. **eTable 8.** Two-way fixed-effects models: changes in mental health before and during the COVID-19 pandemic—complete data analysis. **eTable 9.** Two-way fixed-effects models: interaction effects between changes in mental health and sociodemographic characteristics—complete data analysis. **eTable 10.** Two-way fixed-effects models: changes in mental health before and during the COVID-19 pandemic—models restricted to participants who did not experience COVID-19. **eTable 11.** Two-way fixed-effects models: interaction effects between changes in mental health and sociodemographic characteristics—models restricted to participants who did not experience COVID-19. **eTable 12.** Two-way fixed-effects models: changes in mental health before and during the COVID-19 pandemic (robust standard errors). **eTable 13.** Descriptive statistics of the mental health outcomes from wave 4 to COVID-19 wave 2. **eTable 14.** Two-way fixed-effects models: changes in mental health before (wave 4 to 9) and during the COVID-19 pandemic. **eFigure 3.** Predicted trajectories of depression, poor quality of life, and loneliness before (waves 4 to 9) and during the COVID-19 pandemic (fixed-effects models). **eTable 15.** Comparison of the differences in loneliness and poor quality of life scores between people with and without chronic physical illnesses at wave 9 vs the changes in loneliness and poor quality of life scores before (wave 9) and during the COVID-19 pandemic (wave 2).
eReferences.
