# Supplementary material for: Immediate and Longer-Term Changes in the Mental Health and Well-being of Older Adults in England During the COVID-19 Pandemic
Source: JAMA Psychiatry. 2021 Dec 22;79(2):1–9. doi: 10.1001/jamapsychiatry.2021.3749 (PMC8696687; doi:10.1001/jamapsychiatry.2021.3749)
Supplement: Supplement. — eMethods. eResults. Sensitivity analyses. eTable 1. Comparison of the characteristics of ELSA participants included in the analytical sample vs those not included at wave 9. eTable 2. Descriptive statistics of the mental health outcomes before and during COVID-19. eTable 3. Predicted values of the mental health outcomes before and during COVID-19 (fixed-effects model results). eFigure 1. Predicted values of the mental health outcomes before and during the COVID-19 pandemic. eTable 4. Two-way fixed-effects models: changes in mental health before and during the COVID-19 pandemic. eTable 5. Two-way fixed-effects models: interaction effects between changes in mental health and sociodemographic characteristics. eFigure 2. Fixed-effects models: interaction effects between changes in mental health and sociodemographic characteristics. eTable 6. Two-way fixed-effects models: changes in the total scores of depression and anxiety before and during the COVID-19 pandemic. eTable 7. Two-way fixed-effects models: interaction effects between changes in the total scores of depression and anxiety and sociodemographic characteristics. eTable 8. Two-way fixed-effects models: changes in mental health before and during the COVID-19 pandemic—complete data analysis. eTable 9. Two-way fixed-effects models: interaction effects between changes in mental health and sociodemographic characteristics—complete data analysis. eTable 10. Two-way fixed-effects models: changes in mental health before and during the COVID-19 pandemic—models restricted to participants who did not experience COVID-19. eTable 11. Two-way fixed-effects models: interaction effects between changes in mental health and sociodemographic characteristics—models restricted to participants who did not experience COVID-19. eTable 12. Two-way fixed-effects models: changes in mental health before and during the COVID-19 pandemic (robust standard errors). eTable 13. Descriptive statistics of the mental health outcomes from wave 4 to [file jamapsychiatry-e213749-s001.pdf]

Supplemental Online Content

Zaninotto P, Iob E, Demakakos P, Steptoe A. Immediate and longer-term changes in the mental health and well-being of older adults in England during the COVID-19 pandemic. *JAMA Psychiatry*. Published online December 22, 2021. doi:10.1001/jamapsychiatry.2021.3749

eMethods.
eResults. Sensitivity analyses
eTable 1. Comparison of the characteristics of ELSA participants included in the analytical sample vs those not included at wave 9
eTable 2. Descriptive statistics of the mental health outcomes before and during COVID-19
eTable 3. Predicted values of the mental health outcomes before and during COVID-19 (fixed-effects model results)
eFigure 1. Predicted values of the mental health outcomes before and during the COVID-19 pandemic
eTable 4. Two-way fixed-effects models: changes in mental health before and during the COVID-19 pandemic
eTable 5. Two-way fixed-effects models: interaction effects between changes in mental health and sociodemographic characteristics
eFigure 2. Fixed-effects models: interaction effects between changes in mental health and sociodemographic characteristics
eTable 6. Two-way fixed-effects models: changes in the total scores of depression and anxiety before and during the COVID-19 pandemic
eTable 7. Two-way fixed-effects models: interaction effects between changes in the total scores of depression and anxiety and sociodemographic characteristics
eTable 8. Two-way fixed-effects models: changes in mental health before and during the COVID-19 pandemic—complete data analysis
eTable 9. Two-way fixed-effects models: interaction effects between changes in mental health and sociodemographic characteristics—complete data analysis
eTable 10. Two-way fixed-effects models: changes in mental health before and during the COVID-19 pandemic—models restricted to participants who did not experience COVID-19
eTable 11. Two-way fixed-effects models: interaction effects between changes in mental health and sociodemographic characteristics—models restricted to participants who did not experience COVID-19
eTable 12. Two-way fixed-effects models: changes in mental health before and during the COVID-19 pandemic (robust standard errors)
eTable 13. Descriptive statistics of the mental health outcomes from wave 4 to COVID-19 wave 2
eTable 14. Two-way fixed-effects models: changes in mental health before (wave 4 to 9) and during the COVID-19 pandemic
eFigure 3. Predicted trajectories of depression, poor quality of life, and loneliness before (waves 4 to 9) and during the COVID-19 pandemic (fixed-effects models)

© 2021 Zaninotto P et al. *JAMA Psychiatry*.

eTable 15. Comparison of the differences in loneliness and poor quality of life scores between people with and without chronic physical illnesses at wave 9 vs the changes in loneliness and poor quality of life scores before (wave 9) and during the COVID-19 pandemic (wave 2)
eReferences.

This supplementary material has been provided by the authors to give readers additional information about their work.

© 2021 Zaninotto P et al. *JAMA Psychiatry*.

eMethods.

Outcomes

We focused on the following mental health outcomes: depression, quality of life, loneliness, and anxiety. Depressive symptoms were ascertained using the 8-item Centre for Epidemiological Studies Depression (CESD-8) scale, which measures eight different symptoms of depression (e.g. "felt depressed", "everything I did was an effort", "sleep was restless"). This scale has previously been validated against gold-standard psychiatric interviews with good sensitivity and specificity.[1] A dichotomous (yes/no) response was used for each item, resulting in a total CESD-8 score ranging between zero (no symptoms) and eight (all eight symptoms). We then created a binary variable using a cut-off point of four or more symptoms to identify likely cases of clinical depression, which is equivalent to the conventional threshold of 16 or higher on the full 20-item CESD scale.[2] Quality of life was measured using the 12-item version of the Control, Autonomy, Self-realisation, and Pleasure (CASP) scale, a self-completion questionnaire that has been developed to assess the quality of life and wellbeing of older people. The 12-item version of CASP measures three domains of quality of life, including 'Control and Autonomy' (e.g. "My age prevents me from doing the things I would like to do"), 'Pleasure' (e.g. "I look forward to each day"), and 'Self-realisation' (e.g. "I feel that life is full of opportunities").[3] Each item is scored on a 4-point scale ("Often", "Sometimes", "Not often", "Never"). The resulting item scores were summed to create an index of quality of life where higher scores indicate poorer wellbeing (range: 1-48). Loneliness was assessed using the 3-item revised University of California (UCLA) Loneliness scale[4] ("How often do you feel": 1) "lack of companionship?"; 2) "left out?"; 3) "isolated from others?"), and an additional item asking participants how often they feel lonely. Each question was rated on a 3-point scale (1 = "hardly ever/never"; 2 = "some of the time"; 3 = "often"). The individual item scores were then summed together to produce a total score, with higher values indicating greater loneliness (range: 1-12). Anxiety was measured using the 7-item generalised anxiety disorder scale (GAD-7), which evaluates the presence of various symptoms of generalised anxiety disorder (GAD) (e.g. "Feeling nervous, anxious or on edge", "Not being able to stop or control worrying"). This scale has been shown to be valid and reliable tool for screening for GAD and to assess its severity in both research and clinical practice.[5] Each item is scored on a 4-point scale ("Not at all, "Several days", "More than half the days", "Nearly every day"). We used a total score of 10 or greater as a cut-off point for identifying cases of GAD. To understand the impact of the COVID-19 pandemic on depression, quality of life, and loneliness, we compared the participant's scores at the two COVID-19 waves with those of their most recent assessment before the start of the pandemic (i.e. wave 9). For anxiety, we only compared the change between the two COVID-19 waves, as this scale was not included previously in the regular ELSA survey.

Sociodemographic characteristics

The main sociodemographic characteristics considered in the analysis were: age, sex, wealth, and partnership status. The variable age included three groups: 50-59, 60-74, and 75+ years. Sex was a binary variable (men/women). Wealth was derived from a comprehensive assessment of the participant's economic resources (e.g. financial, housing, and physical wealth) excluding pension wealth, and was categorised into tertiles (1st = lowest wealth; 3rd = highest wealth). Partnership status was a binary variable indicating whether the participant had a partner. We also presented descriptive statistics for the following characteristics: ethnicity (white/other),

© 2021 Zaninotto P et al. *JAMA Psychiatry*.

education (“low” = Compulsory School Leaving/ “medium” = A-levels & College/ “high” = Degree or above), employment status (employed/ retired/ other not working), home tenure (owns outright/ owns with mortgage/ rents), and limiting longstanding illness (no/ yes). Age and partnership status were measured at the first COVID-19 assessment, while wealth, education, employment status and limiting longstanding illness were determined in pre-pandemic assessments (i.e. wave 9 or 8). Further, we derived a binary variable indicating whether the participant had experienced COVID-19 at the first or second COVID-19 wave. The following criteria were applied to identify confirmed or suspected cases of COVID-19: participants were found to be COVID-19 positive on testing, or were hospitalised due to COVID-19, or reported two of the three core symptoms as defined by the UK National Health Service (NHS) (i.e. high temperature, a new continuous cough, and loss of sense of smell or taste).

Missing data

The percentage of missing data in the variables ranged between 0 and 6%. In addition, due to a survey error, for around 75% of the sample the last item of the CESD-8 questionnaire was not administered at the first COVID-19 wave. This type of missing data is classified as missing completely at random (MCAR), and can be dealt efficiently with multiple imputation (MI).[6] We used MI by chained equations with all variables of the analysis included as predictors of the imputation models as well as auxiliary variables. We created twenty imputed datasets, and then pooled the regression estimates across the imputed datasets using Rubin’s rules.[7] The distribution of the variables in the imputed and observed data was similar, suggesting that the MI procedure produced accurate model estimates.

Equations of the fixed-effects regression models

(1) Changes in the outcomes before and during COVID-19

Equation 1

$Y_{it} = \beta_1 X_{it} + \beta_2 Z_{it} + \alpha_i + \lambda_t + u_{it}$,

where Y_{it} is the time-varying dependent variable for individual *i* at time *t*; X_{it} is the first COVID-19 binary indicator (i.e. wave 9 vs COVID-19 w1); Z_{it} is the second COVID-19 binary indicator (i.e. wave 9 vs COVID-19 w2); α_i represents the individual fixed-effects; λ_t represents the time fixed-effects; and u_{it} is the error term.

(2) Interaction effects with sociodemographic factors

(2.1) Individual interaction effects

Equation 2

$Y_{it} = \beta_1 X_{it} + \beta_2 X_{it} S_i + \alpha_i + \lambda_t + u_{it}$,

where X_{it} is the COVID-19 binary indicator (i.e. wave 9 vs COVID-19 wave 1/2) and $X_{it}S_i$ is the interaction term between the COVID-19 indicator and one time-invariant sociodemographic factor (i.e. sex, age, partnership status, or wealth).

© 2021 Zaninotto P et al. *JAMA Psychiatry*.

(2.2) Mutually adjusted interaction effects

Equation 3

$Y_{it} = \beta_1 X_{it} + \beta_2 X_{it} S_i + \beta_3 X_{it} T_i + \beta_4 X_{it} Z_i + \beta_5 X_{it} W_i + \alpha_i + \lambda_t + u_{it}$,

where X_{it} is the COVID-19 binary indicator (wave 9 vs COVID-19 wave 1/2); $X_{it}S_i$ is the interaction term with the first time-invariant sociodemographic factor (i.e. sex); $X_{it}T_i$ is the interaction term with the second time-invariant sociodemographic factor (i.e. age); $X_{it}Z_i$ is the interaction term with the third time-invariant sociodemographic factor (i.e. partnership); and $X_{it}W_i$ is the interaction term with the fourth time-invariant sociodemographic factor (i.e. wealth).

Note. Fixed-effects models do not estimate regression coefficients for time-invariant independent variables as their effects are controlled for by the individual fixed-effects.

Sensitivity analyses

First, we tested changes in depression and anxiety during the COVID-19 pandemic and variations with sociodemographic factors using the total CESD-8 and GAD-7 scores, rather than the binary scores representing cases of depression and anxiety. Second, we reran all models presented in the main imputed data analysis using the sample of participants with complete data on all variables. Third, we restricted the main analyses to participants who did not experience COVID-19 at either the first or second COVID-19 wave. Fourth, the changes in the mental health outcomes before and during COVID-19 were estimated using fixed-effects models with robust standard errors to account for potential heteroskedasticity and autocorrelation. Lastly, we examined changes in depression, quality of life, and loneliness before and during COVID-19 accounting for pre-pandemic trends in mental health from wave 4 (2008/09) to 9 (2018/19).

© 2021 Zaninotto P et al. *JAMA Psychiatry*.

eResults. Sensitivity analyses

First, we tested changes in depression and anxiety during the COVID-19 pandemic and variations with sociodemographic factors using the total CESD-8 and GAD-7 scores, rather than the binary scores representing cases of depression and anxiety. The results mirrored those of the analysis with the binary scores (SI Appendix – sTable6 and sTable7). The change in the total score of depression before and during COVID-19 was smaller than the change in the binary depression score, but still considerably larger than the change observed for the total scores of quality of life and loneliness. The changes in the total and binary scores of anxiety were broadly similar (SI Appendix – sTable4 and sTable6). It is also worth noting that the predicted probabilities of depression and anxiety derived from the fixed-effects linear probability models were all within the range of 0-1 (see Figures 1 and 3), thereby providing further evidence for the adequacy of these models. Second, we reran all models presented in the main imputed data analysis using the sample of participants with complete data on all variables. The pattern of changes in mental health before and during COVID-19 and interaction effects with sociodemographic factors aligned closely with the results found in the main imputed analysis (SI Appendix – sTable8 and sTable9). Third, we restricted the main analyses to participants who did not experience COVID-19 at either the first or second COVID-19 wave. A total of 5·4% experienced COVID-19 on the basis of testing, hospitalisation, or symptoms, leaving 4867 (94·6%) participants in these analyses. There were no substantial changes from the primary analyses in the magnitude and statistical significance of the associations (SI Appendix – sTable10 and sTable11). Fourth, the changes in the mental health outcomes before and during COVID-19 were estimated using fixed-effects models with robust standard errors to account for potential heteroskedasticity and autocorrelation. The results were almost identical to those found in the main analysis (sTable12). Lastly, we examined changes in depression, quality of life, and loneliness before and during COVID-19 accounting for pre-pandemic trends in mental health from wave 4 (2008/09) to 9 (2018/19). Anxiety was not included in this sensitivity analysis since the GAD-7 scale was not administered in the regular ELSA survey. The observed percentages/means of the mental health outcomes from wave 4 through to COVID-19 wave 2 are presented in sTable13. The estimated changes in mental health during COVID-19 (wave 1 and 2) versus before (wave 9) adjusted for earlier trends (wave 4 to 8) aligned closely with those observed in the main analyses (sTable14, Model 1). sFigure3 shows the estimated trajectories of depression, poor quality of life, and loneliness from wave 4 to COVID-19 wave 2. As can be seen, the increase in psychological distress during COVID-19 occurred against a slight downward trend in all mental health outcomes over the preceding years. Of note, the estimated prevalence of depression in the sample during COVID-19 was also considerably larger than the average depression prevalence over the preceding 11/12 years (sTable14, Model 2), providing corroborative evidence of marked increases in depressive symptoms during COVID-19.

© 2021 Zaninotto P et al. *JAMA Psychiatry*.

eTable 1. Comparison of the characteristics of ELSA participants included in the analytical sample vs those not included at wave 9

	**Non-included (N=3590)**	**Included (N=5146)**	**p-value**
**Age**			0.004
Mean (SD)	67.566 (11.275)	68.207 (9.045)	
Range	50.000 - 90.000	50.000 - 90.000	
**Sex**			0.016
Male	1639 (45.7%)	2215 (43.0%)	
Female	1951 (54.3%)	2931 (57.0%)	
**Ethnicity**			< 0.001
White	3343 (93.3%)	4955 (96.3%)	
Non-White	241 (6.7%)	189 (3.7%)	
**Work status**			< 0.001
Retired	1845 (51.4%)	3305 (64.2%)	
Employed	1323 (36.9%)	1437 (27.9%)	
Other not working	422 (11.8%)	404 (7.9%)	
**Wealth**			0.014
Mean (SD)	189634.083 (525585.754)	217683.739 (512556.189)	
Range	-55000.000 - 12416100.000	-100900.000 - 12416100.000	
**Self-rated health**			< 0.001
Excellent	404 (12.9%)	629 (12.4%)	
Very good	718 (23.0%)	1653 (32.6%)	
Good	1006 (32.2%)	1699 (33.6%)	
Fair	664 (21.3%)	794 (15.7%)	
Poor	332 (10.6%)	289 (5.7%)	

Note. *P-values obtained from t-tests (continuous variables) or chi-square tests (categorical variables); SD = standard deviation.*

© 2021 Zaninotto P et al. *JAMA Psychiatry*.

eTable 2. Descriptive statistics of the mental health outcomes before and during COVID-19

	**Imputed data, weighted**		
	**Before COVID-19 (Wave 9)**	**During COVID-19 (Wave 1)**	**During COVID-19 (Wave 2)**
**Elevated depressive symptoms (CESD-8 ≥ 4)**			
No	87.50%	77.40%	71.50%
Yes	12.50%	22.60%	28.50%
**Poor Quality of Life (CASP-12)**			
Mean (SD)	21.602 (6.283)	22.529 (6.526)	23.062 (6.670)
Range	1.000 - 48.000	12.000 - 47.000	9.000 - 48.000
**Loneliness**			
Mean (SD)	5.496 (2.026)	5.648 (2.065)	5.748 (2.172)
Range	1.000 - 12.000	2.000 - 12.000	3.000 - 12.000
**Anxiety (Gad-7 ≥ 10)**			
No	NA	90.60%	89.10%
Yes	NA	9.40%	10.90%
Note. *ELSA COVID-19 longitudinal sample (N=5146); SD = standard deviation; NA = Not Available.*			

© 2021 Zaninotto P et al. *JAMA Psychiatry*.

eTable 3. Predicted values of the mental health outcomes before and during COVID-19 (fixed-effects model results)

**Outcome**	**Wave**	**Predicted values**	**Observed values**
		**%**	**%**
Depression	Before COVID-19	12.5	12.5
	COVID-19 w1	22.6	22.6
	COVID-19 w2	28.5	28.5
Anxiety	COVID-19 w1	9.4	9.4
	COVID-19 w2	10.9	10.9
		**Mean (s.e.)**	**Mean(SD)**
Poor QoL (total score)	Before COVID-19	21.6 (0.06)	21.6 (6.28)
	COVID-19 w1	22.5 (0.05)	22.5 (6.53)
	COVID-19 w2	23.1(0.05)	23.1 (6.67)
Loneliness (total score)	Before COVID-19	5.5 (0.02)	5.5 (2.03)
	COVID-19 w1	5.6 (0.02)	5.6 (2.07)
	COVID-19 w2	5.7 (0.02)	5.7 (2.17)
Note. *ELSA COVID-19 longitudinal sample (N=5,146); weighted pooled estimates from two-way fixed-effects linear models across 20 imputed datasets. SE = standard error. SD = standard deviation. QoL = quality of life.*			

© 2021 Zaninotto P et al. *JAMA Psychiatry*.

eFigure 1. Predicted values of the mental health outcomes before and during the COVID-19 pandemic

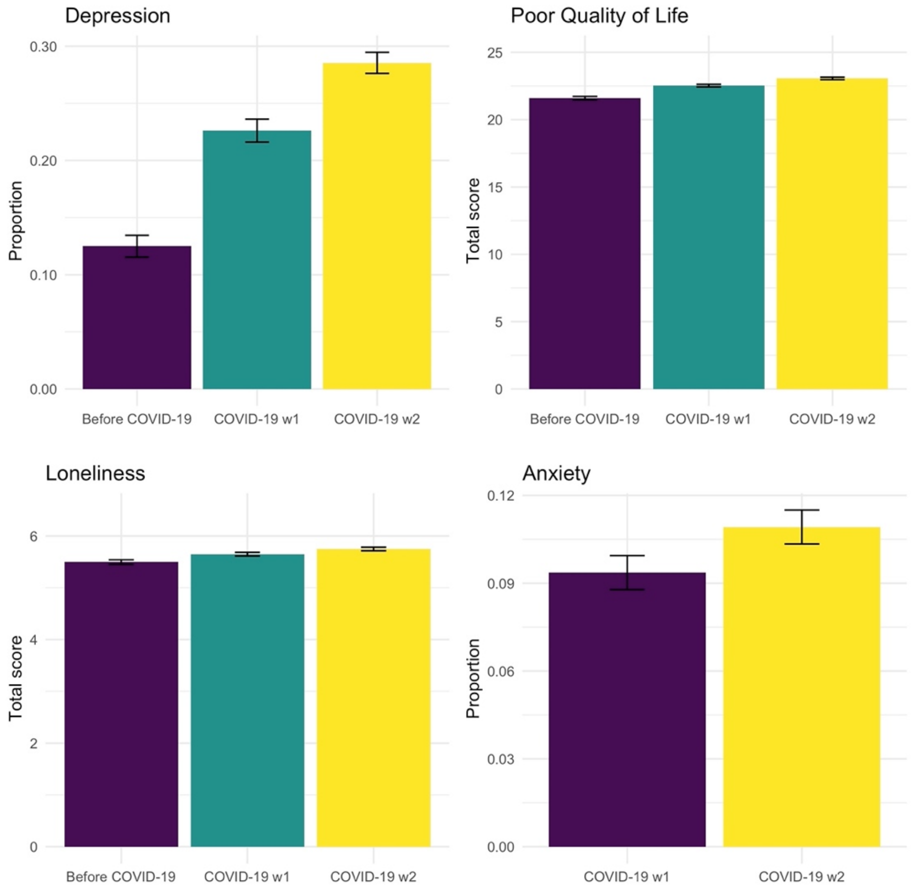

Note. *ELSA COVID-19 longitudinal sample (N=5146); weighted pooled estimates from two-way fixed-effects linear models across 20 imputed datasets.*

© 2021 Zaninotto P et al. *JAMA Psychiatry*.

eTable 4. Two-way fixed-effects models: changes in mental health before and during the COVID-19 pandemic

	B	SE	p-value	CI (lower)	CI (upper)	% change[a]
**Outcome: Depression**						
Intercept	0.125	0.005	**0.000**	0.115	0.134	
Change before vs during COVID-19 (average)	0.131	0.007	**0.000**	0.117	0.145	104.75
Intercept	0.125	0.005	**0.000**	0.115	0.134	
Change before vs during COVID-19 (w1)	0.101	0.008	**0.000**	0.085	0.117	81.02
Change before vs during COVID-19 (w2)	0.160	0.008	**0.000**	0.144	0.177	128.48
Intercept	0.226	0.005	**0.000**	0.216	0.236	
Change COVID-19 w1 vs w2	0.059	0.008	**0.000**	0.043	0.075	26.22
**Outcome: Poor Quality of Life**						
Intercept	21.602	0.062	**0.000**	21.480	21.724	
Change before vs during COVID-19 (average)	1.194	0.090	**0.000**	1.018	1.370	5.53
Intercept	21.602	0.062	**0.000**	21.480	21.724	
Change before vs during COVID-19 (w1)	0.927	0.099	**0.000**	0.732	1.122	4.29
Change before vs during COVID-19 (w2)	1.461	0.096	**0.000**	1.272	1.649	6.76
Intercept	22.529	0.049	**0.000**	22.432	22.626	
Change COVID-19 w1 vs w2	0.534	0.078	**0.000**	0.381	0.686	2.37
**Outcome: Loneliness**						
Intercept	5.496	0.022	**0.000**	5.452	5.540	
Change before vs during COVID-19 (average)	0.202	0.032	**0.000**	0.140	0.265	3.68
Intercept	5.496	0.022	**0.000**	5.452	5.540	
Change before vs during COVID-19 (w1)	0.152	0.035	**0.000**	0.083	0.221	2.77
Change before vs during COVID-19 (w2)	0.252	0.035	**0.000**	0.183	0.322	4.59
Intercept	5.648	0.018	**0.000**	5.613	5.683	
Change COVID-19 w1 vs w2	0.100	0.029	**0.001**	0.043	0.157	1.77
**Outcome: Anxiety**						
Intercept	0.094	0.003	**0.000**	0.088	0.099	
Change COVID-19 w1 vs w2	0.016	0.006	**0.008**	0.004	0.027	16.62

Note. *ELSA COVID-19 longitudinal sample (N=5146); weighted pooled estimates across 20 imputed datasets; p-values highlighted in bold are statistically significant at the 95% confidence level. [a] Calculated as change score (i.e. slope) divided by baseline value (i.e. intercept) and multiplied by 100%.*

© 2021 Zaninotto P et al. *JAMA Psychiatry*.

eTable 5. Two-way fixed-effects models: interaction effects between changes in mental health and sociodemographic characteristics

		Outcome: Depression					Outcome: Poor Quality of Life					Outcome: Loneliness					Outcome: Anxiety				
**Sociodemographic characteristics**		**B**	**SE**	**p-value**	**CI (lower)**	**CI (upper)**	**B**	**SE**	**p-value**	**CI (lower)**	**CI (upper)**	**B**	**SE**	**p-value**	**CI (lower)**	**CI (upper)**	**B**	**SE**	**p-value**	**CI (lower)**	**CI (upper)**
**a. Individual interactions**																					
Age group	Change	0.149	0.015	**0.000**	0.119	0.180	1.348	0.195	**0.000**	0.967	1.730	0.251	0.073	**0.001**	0.108	0.394	0.016	0.006	**0.008**	0.004	0.027
	Change*50-59	ref					ref					ref					ref				
	Change*60-74	-0.017	0.019	0.367	-0.053	0.020	-0.319	0.225	0.157	-0.760	0.123	-0.113	0.083	0.174	-0.275	0.050	-0.017	0.016	0.295	-0.048	0.014
	Change*75 and over	-0.047	0.020	**0.021**	-0.086	-0.007	-0.055	0.261	0.834	-0.566	0.457	0.006	0.093	0.948	-0.177	0.189	-0.018	0.017	0.281	-0.051	0.015
Sex	Change	0.113	0.011	**0.000**	0.092	0.134	0.759	0.143	**0.000**	0.480	1.039	0.073	0.046	0.115	-0.018	0.164	0.000	0.000		0.000	0.000
	Change*Men	ref					ref					ref					ref				
	Change*Women	0.034	0.015	**0.022**	0.005	0.063	0.821	0.191	**0.000**	0.447	1.196	0.245	0.064	**0.000**	0.120	0.369	0.024	0.012	**0.043**	0.001	0.047
Wealth (tertiles)	Change	0.123	0.011	**0.000**	0.102	0.145	1.465	0.124	**0.000**	1.221	1.708	0.262	0.044	**0.000**	0.176	0.348	0.000	0.000		0.000	0.000
	Change*1st tertile	0.018	0.017	0.314	-0.017	0.052	-0.417	0.207	**0.044**	-0.823	-0.011	-0.065	0.073	0.374	-0.209	0.079	-0.015	0.014	0.280	-0.042	0.012
	Change*2nd tertile	-0.001	0.015	0.959	-0.031	0.030	-0.309	0.189	0.103	-0.680	0.062	-0.106	0.067	0.115	-0.237	0.026	0.001	0.012	0.907	-0.023	0.026
	Change*3rd tertile	ref					ref					ref					ref				
Partnership	Change	0.126	0.008	**0.000**	0.111	0.142	1.222	0.102	**0.000**	1.022	1.422	0.146	0.035	**0.000**	0.078	0.215	0.000	0.000		0.000	0.000
	Change*Partnered	ref					ref					ref					ref				
	Change*Non-partnered	0.020	0.019	0.299	-0.017	0.056	-0.116	0.213	0.586	-0.534	0.302	0.228	0.080	**0.005**	0.070	0.386	-0.022	0.013	0.097	-0.049	0.004
**b. Mutually adjusted interactions**																					
	Change	0.124	0.021	**0.000**	0.084	0.165	1.302	0.235	**0.000**	0.841	1.763	0.205	0.086	**0.018**	0.035	0.374	0.000	0.000		0.000	0.000
Age group	Change*50-59	ref					ref					ref					ref				
	Change*60-74	-0.017	0.019	0.370	-0.054	0.020	-0.386	0.226	0.087	-0.828	0.057	-0.143	0.083	0.084	-0.306	0.019	-0.018	0.016	0.246	-0.049	0.013
	Change*75 and over	-0.053	0.020	**0.007**	-0.092	-0.014	-0.121	0.275	0.659	-0.660	0.418	-0.072	0.096	0.455	-0.261	0.117	-0.017	0.017	0.296	-0.050	0.015
Sex	Change*Men	ref					ref					ref					ref				
	Change*Women	0.033	0.015	**0.026**	0.004	0.063	0.856	0.194	**0.000**	0.476	1.236	0.235	0.064	**0.000**	0.110	0.360	0.027	0.012	**0.026**	0.003	0.051
Wealth (tertiles)	Change*1st tertile	0.008	0.018	0.659	-0.027	0.043	-0.528	0.214	**0.013**	-0.947	-0.110	-0.145	0.074	**0.050**	-0.290	0.000	-0.017	0.014	0.236	-0.044	0.011
	Change*2nd tertile	-0.002	0.016	0.906	-0.033	0.029	-0.351	0.189	0.064	-0.722	0.020	-0.137	0.067	**0.043**	-0.269	-0.004	0.001	0.012	0.913	-0.023	0.026
	Change*3rd tertile	ref					ref					ref					ref				
Partnership	Change*Partnered	ref					ref					ref					ref				
	Change*Non-partnered	0.025	0.019	0.194	-0.013	0.062	-0.122	0.227	0.591	-0.567	0.323	0.230	0.084	**0.006**	0.066	0.394	-0.020	0.014	0.162	-0.047	0.008

© 2021 Zaninotto P et al. *JAMA Psychiatry*.

Note. *ELSA COVID-19 longitudinal sample (N=5146); weighted pooled estimates across 20 imputed datasets; p-values highlighted in bold are statistically significant at the 95% confidence level; interaction effects of sociodemographic characteristics with change before and/or during COVID-19 (average).*

© 2021 Zaninotto P et al. *JAMA Psychiatry*.

eFigure 2. Fixed-effects models: interaction effects between changes in mental health and sociodemographic characteristics

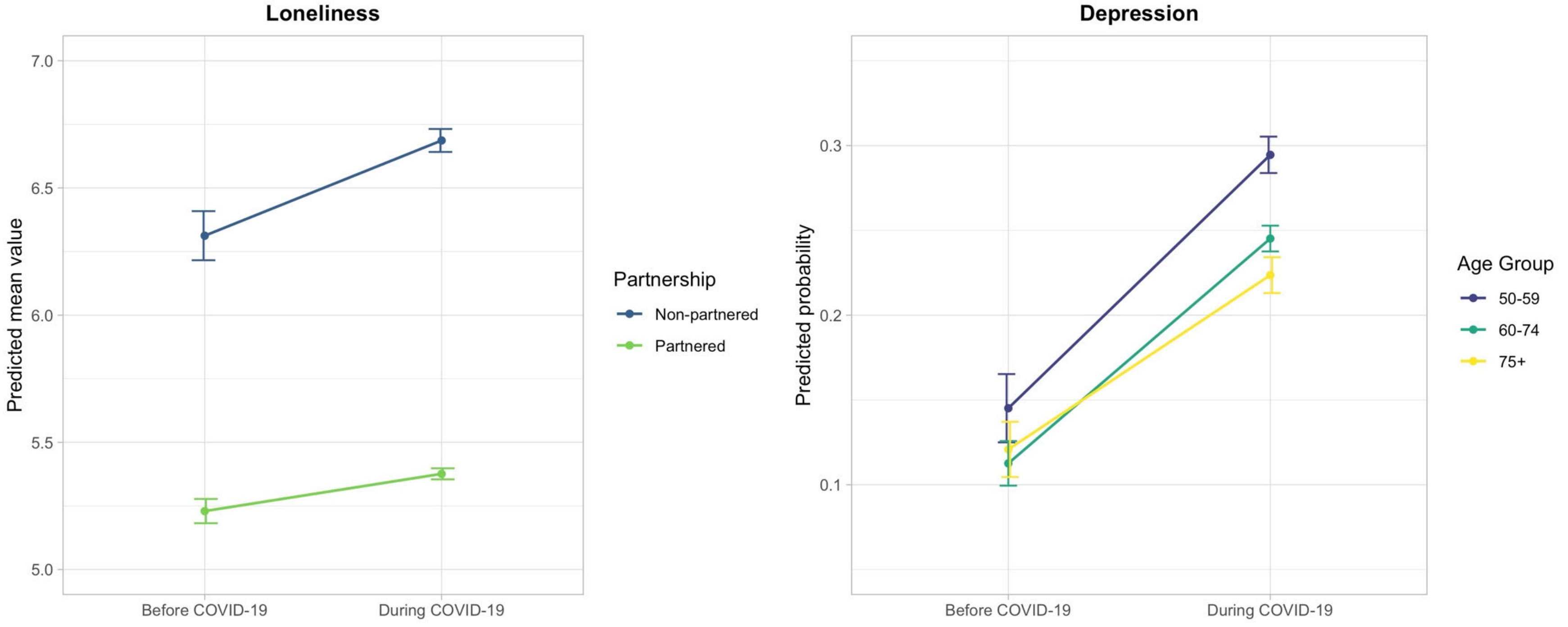

Note. *ELSA COVID-19 longitudinal sample (N=5146); predicted values of the outcomes by sociodemographic characteristics, derived from mutually adjusted two-way fixed-effects linear models; weighted pooled estimates across 20 imputed datasets.*

© 2021 Zaninotto P et al. *JAMA Psychiatry*.

eTable 6. Two-way fixed-effects models: changes in the total scores of depression and anxiety before and during the COVID-19 pandemic

	B	SE	p-value	CI (lower)	CI (upper)	% change[a]
**Outcome: Depression**						
Change before vs during COVID-19 (average)	0.774	0.037	**0.000**	0.702	0.846	55.01
Change before vs during COVID-19 (w1)	0.638	0.040	**0.000**	0.559	0.717	45.33
Change before vs during COVID-19 (w2)	0.911	0.041	**0.000**	0.831	0.991	64.68
Change COVID-19 w1 vs w2	0.272	0.035	**0.000**	0.204	0.341	13.31
**Outcome: Anxiety**						
Change COVID-19 w1 vs w2	0.369	0.070	**0.000**	0.230	0.507	11.22

Note. *ELSA COVID-19 longitudinal sample (N=5146); weighted pooled estimates across 20 imputed datasets; p-values highlighted in bold are statistically significant at the 95% confidence level.* [a] *Calculated as change score (i.e. slope) divided by baseline value (i.e. intercept) and multiplied by 100%.*

© 2021 Zaninotto P et al. *JAMA Psychiatry*.

eTable 7. Two-way fixed-effects models: interaction effects between changes in the total scores of depression and anxiety and sociodemographic characteristics

		**Outcome: Depression**					**Outcome: Anxiety**				
**Sociodemographic characteristics**		**B**	**SE**	**p-value**	**CI (lower)**	**CI (upper)**	**B**	**SE**	**p-value**	**CI (lower)**	**CI (upper)**
**a. Individual interactions**											
Age group	Change	0.919	0.078	**0.000**	0.767	1.071	0.510	0.160	**0.001**	0.197	0.823
	Change*50-59	ref					ref				
	Change*60-74	-0.132	0.094	0.160	-0.315	0.052	-0.183	0.185	0.324	-0.546	0.181
	Change*75 and over	-0.363	0.098	**0.000**	-0.555	-0.172	-0.254	0.192	0.185	-0.630	0.122
Sex	Change	0.678	0.056	**0.000**	0.568	0.788	0.263	0.108	**0.015**	0.051	0.475
	Change*Men	ref					ref				
	Change*Women	0.183	0.074	**0.014**	0.037	0.329	0.200	0.142	0.159	-0.078	0.479
Wealth (tertiles)	Change	0.722	0.055	**0.000**	0.615	0.829	0.398	0.106	**0.000**	0.190	0.607
	Change*1st tertile	0.094	0.088	0.285	-0.078	0.266	-0.054	0.168	0.747	-0.383	0.275
	Change*2nd tertile	0.041	0.079	0.605	-0.114	0.195	-0.022	0.152	0.887	-0.320	0.277
	Change*3rd tertile	ref					ref				
Partnership	Change	0.739	0.040	**0.000**	0.661	0.818	0.366	0.080	**0.000**	0.209	0.523
	Change*Partnered	ref					ref				
	Change*Non-partnered	0.143	0.094	0.130	-0.042	0.328	0.009	0.168	0.957	-0.320	0.339
**b. Mutually adjusted interactions**											
	Change	0.771	0.100	**0.000**	0.575	0.968	0.461	0.199	**0.020**	0.071	0.851
Age group	Change*50-59	ref					ref				
	Change*60-74	-0.140	0.094	0.139	-0.325	0.045	-0.202	0.187	0.280	-0.569	0.165
	Change*75 and over	-0.422	0.099	**0.000**	-0.615	-0.229	-0.292	0.193	0.131	-0.671	0.087
Sex	Change*Men	ref					ref				
	Change*Women	0.183	0.076	**0.016**	0.035	0.332	0.212	0.146	0.146	-0.074	0.498
Wealth (tertiles)	Change*1st tertile	0.023	0.089	0.799	-0.152	0.198	-0.112	0.174	0.518	-0.453	0.228
	Change*2nd tertile	0.033	0.079	0.675	-0.122	0.189	-0.033	0.154	0.828	-0.335	0.268
	Change*3rd tertile	ref					ref				
Partnership	Change*Partnered	ref					ref				
	Change*Non-partnered	0.200	0.098	**0.041**	0.008	0.391	0.053	0.175	0.762	-0.290	0.396

Note. *ELSA COVID-19 longitudinal sample (N=5146); weighted pooled estimates across 20 imputed datasets; p-values highlighted in bold are statistically significant at the 95% confidence level.*

© 2021 Zaninotto P et al. *JAMA Psychiatry*.

eTable 8. Two-way fixed-effects models: changes in mental health before and during the COVID-19 pandemic—complete data analysis

	B	SE	p-value	CI (lower)	CI (upper)
**Outcome: Depression (N=5047)**					
Change before vs during COVID-19 (average)	0.130	0.007	**0.000**	0.116	0.144
Change before vs during COVID-19 (w1)	0.100	0.007	**0.000**	0.085	0.115
Change before vs during COVID-19 (w2)	0.160	0.008	**0.000**	0.144	0.177
Change COVID-19 w1 vs w2	0.061	0.007	**0.000**	0.046	0.075
**Outcome: Poor Quality of Life (N=5047)**					
Change before vs during COVID-19 (average)	1.194	0.083	**0.000**	1.031	1.357
Change before vs during COVID-19 (w1)	0.927	0.093	**0.000**	0.744	1.110
Change before vs during COVID-19 (w2)	1.461	0.090	**0.000**	1.284	1.638
Change COVID-19 w1 vs w2	0.534	0.078	**0.000**	0.381	0.686
**Outcome: Loneliness (N=5047)**					
Change before vs during COVID-19 (average)	0.202	0.029	**0.000**	0.145	0.260
Change before vs during COVID-19 (w1)	0.152	0.033	**0.000**	0.088	0.217
Change before vs during COVID-19 (w2)	0.252	0.033	**0.000**	0.188	0.317
Change COVID-19 w1 vs w2	0.100	0.029	**0.001**	0.043	0.157
**Outcome: Anxiety (N=5106)**					
Change COVID-19 w1 vs w2	0.016	0.006	**0.008**	0.004	0.027
Note. *Complete data analysis; weighted estimates; p-values highlighted in bold are statistically significant at the 95% confidence level.*					

© 2021 Zaninotto P et al. *JAMA Psychiatry*.

eTable 9. Two-way fixed-effects models: interaction effects between changes in mental health and sociodemographic characteristics—complete data analysis

		Outcome: Depression (N=5047)					Outcome: Poor Quality of Life (N=5047)					Outcome: Loneliness (N=5047)					Outcome: Anxiety (N=5106)				
**Sociodemographic characteristics**		**B**	**SE**	**p-value**	**CI (lower)**	**CI (upper)**	**B**	**SE**	**p-value**	**CI (lower)**	**CI (upper)**	**B**	**SE**	**p-value**	**CI (lower)**	**CI (upper)**	**B**	**SE**	**p-value**	**CI (lower)**	**CI (upper)**
**a. Individual interactions**																					
Age group	Change	0.149	0.015	**0.000**	0.120	0.178	1.350	0.171	**0.000**	1.016	1.685	0.251	0.065	**0.000**	0.123	0.379	0.027	0.014	0.053	0.000	0.055
	Change*50-59	ref					ref					ref					ref				
	Change*60-74	-0.017	0.018	0.326	-0.052	0.017	-0.322	0.202	0.111	-0.718	0.074	-0.113	0.075	0.132	-0.260	0.034	-0.017	0.016	0.293	-0.048	0.014
	Change*75 and over	-0.047	0.019	**0.014**	-0.084	-0.010	-0.057	0.242	0.815	-0.531	0.418	0.006	0.085	0.942	-0.160	0.172	-0.018	0.017	0.279	-0.051	0.015
Sex	Change	0.112	0.010	**0.000**	0.092	0.133	0.759	0.132	**0.000**	0.500	1.019	0.073	0.042	0.086	-0.010	0.155	0.003	0.009	0.750	-0.015	0.020
	Change*Men	ref					ref					ref					ref				
	Change*Women	0.033	0.014	**0.018**	0.006	0.061	0.821	0.168	**0.000**	0.491	1.151	0.245	0.059	**0.000**	0.130	0.360	0.024	0.012	**0.042**	0.001	0.047
Wealth (tertiles)	Change	0.123	0.010	**0.000**	0.103	0.143	1.466	0.115	**0.000**	1.242	1.691	0.262	0.039	**0.000**	0.185	0.338	0.022	0.008	**0.008**	0.006	0.037
	Change*1st tertile	0.018	0.017	0.287	-0.015	0.050	-0.421	0.191	**0.028**	-0.795	-0.046	-0.066	0.067	0.325	-0.197	0.065	-0.015	0.014	0.277	-0.041	0.012
	Change*2nd tertile	-0.001	0.015	0.955	-0.029	0.028	-0.310	0.175	0.076	-0.652	0.032	-0.106	0.061	0.083	-0.225	0.014	0.001	0.012	0.905	-0.022	0.025
	Change*3rd tertile	ref					ref					ref					ref				
Partnership	Change	0.125	0.008	**0.000**	0.111	0.140	1.222	0.094	**0.000**	1.039	1.406	0.146	0.032	**0.000**	0.084	0.208	0.021	0.007	**0.002**	0.008	0.035
	Change*Partnered	ref					ref					ref					ref				
	Change*Non-partnered	0.020	0.018	0.286	-0.016	0.055	-0.116	0.202	0.567	-0.512	0.281	0.229	0.076	**0.002**	0.081	0.377	-0.022	0.013	0.097	-0.049	0.004
**b. Mutually adjusted interactions**																					
	Change	0.124	0.019	**0.000**	0.088	0.161	1.306	0.214	**0.000**	0.886	1.726	0.204	0.074	**0.006**	0.060	0.349	0.025	0.016	0.126	-0.007	0.057
Age group	Change*50-59	ref					ref					ref					ref				
	Change*60-74	-0.017	0.018	0.327	-0.052	0.017	-0.390	0.205	0.057	-0.792	0.012	-0.144	0.074	0.054	-0.290	0.003	-0.018	0.016	0.244	-0.049	0.013
	Change*75 and over	-0.054	0.019	**0.004**	-0.091	-0.017	-0.124	0.259	0.632	-0.631	0.383	-0.072	0.088	0.414	-0.245	0.101	-0.018	0.017	0.293	-0.050	0.015
Sex	Change*Men	ref					ref					ref					ref				
	Change*Women	0.033	0.014	**0.021**	0.005	0.061	0.856	0.171	**0.000**	0.520	1.192	0.236	0.059	**0.000**	0.120	0.351	0.027	0.012	**0.025**	0.003	0.051
Wealth (tertiles)	Change*1st tertile	0.008	0.017	0.640	-0.025	0.041	-0.532	0.199	**0.007**	-0.922	-0.143	-0.145	0.067	**0.030**	-0.277	-0.014	-0.017	0.014	0.232	-0.044	0.011
	Change*2nd tertile	-0.002	0.015	0.898	-0.031	0.027	-0.352	0.174	**0.043**	-0.694	-0.011	-0.137	0.061	**0.025**	-0.256	-0.017	0.001	0.012	0.911	-0.023	0.025
	Change*3rd tertile	ref					ref					ref					ref				
Partnership	Change*Partnered	ref					ref					ref					ref				
	Change*Non-partnered	0.025	0.019	0.183	-0.012	0.061	-0.121	0.215	0.574	-0.542	0.301	0.231	0.079	**0.003**	0.076	0.386	-0.020	0.014	0.161	-0.047	0.008

Note. *Complete data analysis; weighted estimates; p-values highlighted in bold are statistically significant at the 95% confidence level. Interaction effects of sociodemographic characteristics with change before and during COVID-19 (average).*

© 2021 Zaninotto P et al. *JAMA Psychiatry*.

eTable 10. Two-way fixed-effects models: changes in mental health before and during the COVID-19 pandemic—models restricted to participants who did not experience COVID-19

	B	SE	p-value	CI (lower)	CI (upper)
**Outcome: Depression**					
Change before vs during COVID-19 (average)	0.129	0.007	**0.000**	0.114	0.143
Change before vs during COVID-19 (w1)	0.100	0.008	**0.000**	0.084	0.117
Change before vs during COVID-19 (w2)	0.157	0.009	**0.000**	0.140	0.174
Change COVID-19 w1 vs w2	0.057	0.008	**0.000**	0.041	0.073
**Outcome: Poor Quality of Life**					
Change before vs during COVID-19 (average)	1.151	0.088	**0.000**	0.980	1.323
Change before vs during COVID-19 (w1)	0.885	0.098	**0.000**	0.693	1.078
Change before vs during COVID-19 (w2)	1.417	0.094	**0.000**	1.233	1.601
Change COVID-19 w1 vs w2	0.532	0.078	**0.000**	0.378	0.685
**Outcome: Loneliness**					
Change before vs during COVID-19 (average)	0.170	0.032	**0.000**	0.107	0.233
Change before vs during COVID-19 (w1)	0.115	0.035	**0.001**	0.046	0.185
Change before vs during COVID-19 (w2)	0.225	0.035	**0.000**	0.156	0.294
Change COVID-19 w1 vs w2	0.110	0.029	**0.000**	0.052	0.167
**Outcome: Anxiety**					
Change COVID-19 w1 vs w2	0.015	0.006	**0.016**	0.003	0.026
Note. *ELSA COVID-19 longitudinal sample (N=4867); weighted pooled estimates across 20 imputed datasets; p-values highlighted in bold are statistically significant at the 95% confidence level.*					

© 2021 Zaninotto P et al. *JAMA Psychiatry*.

eTable 11. Two-way fixed-effects models: interaction effects between changes in mental health and sociodemographic characteristics—models restricted to participants who did not experience COVID-19

		Outcome: Depression					Outcome: Poor Quality of Life					Outcome: Loneliness					Outcome: Anxiety				
**Sociodemographic characteristics**		**B**	**SE**	**p-value**	**CI (lower)**	**CI (upper)**	**B**	**SE**	**p-value**	**CI (lower)**	**CI (upper)**	**B**	**SE**	**p-value**	**CI (lower)**	**CI (upper)**	**B**	**SE**	**p-value**	**CI (lower)**	**CI (upper)**
**a. Individual interactions**																					
Age group	Change	0.147	0.016	**0.000**	0.115	0.179	1.347	0.197	**0.000**	0.961	1.733	0.177	0.075	**0.018**	0.030	0.324	0.024	0.015	0.113	-0.006	0.053
	Change*50-59	ref					ref					ref					ref				
	Change*60-74	-0.018	0.019	0.352	-0.055	0.020	-0.365	0.228	0.109	-0.811	0.082	-0.047	0.084	0.577	-0.213	0.118	-0.011	0.017	0.488	-0.044	0.021
	Change*75 and over	-0.043	0.020	**0.035**	-0.083	-0.003	-0.130	0.251	0.604	-0.622	0.362	0.058	0.093	0.532	-0.125	0.242	-0.016	0.018	0.351	-0.051	0.018
Sex	Change	0.112	0.011	**0.000**	0.091	0.133	0.651	0.138	**0.000**	0.382	0.921	0.048	0.047	0.306	-0.044	0.140	0.006	0.009	0.537	-0.012	0.024
	Change*Men	ref					ref					ref					ref				
	Change*Women	0.031	0.015	**0.035**	0.002	0.061	0.945	0.178	**0.000**	0.596	1.293	0.230	0.064	**0.000**	0.105	0.356	0.017	0.012	0.167	-0.007	0.041
Wealth (tertiles)	Change	0.122	0.011	**0.000**	0.100	0.144	1.459	0.130	**0.000**	1.204	1.713	0.255	0.045	**0.000**	0.166	0.343	0.022	0.008	**0.008**	0.006	0.038
	Change*1st tertile	0.012	0.018	0.496	-0.023	0.047	-0.547	0.208	**0.009**	-0.954	-0.139	-0.131	0.075	0.082	-0.279	0.017	-0.018	0.014	0.213	-0.046	0.010
	Change*2nd tertile	0.004	0.016	0.783	-0.027	0.036	-0.261	0.195	0.179	-0.643	0.120	-0.098	0.067	0.139	-0.229	0.032	0.002	0.012	0.899	-0.022	0.025
	Change*3rd tertile	ref					ref					ref					ref				
Partnership	Change	0.125	0.008	**0.000**	0.109	0.141	1.174	0.099	**0.000**	0.980	1.367	0.128	0.037	**0.000**	0.057	0.200	0.019	0.007	**0.008**	0.005	0.033
	Change*Partnered	ref					ref					ref					ref				
	Change*Non-partnered	0.015	0.019	0.410	-0.021	0.052	-0.090	0.211	0.671	-0.502	0.323	0.169	0.077	**0.028**	0.018	0.319	-0.017	0.014	0.199	-0.044	0.009
**b. Mutually adjusted interactions**																					
	Change	0.125	0.021	**0.000**	0.084	0.166	1.290	0.254	**0.000**	0.792	1.787	0.164	0.090	0.068	-0.012	0.341	0.025	0.017	0.145	-0.009	0.059
Age group	Change*50-59	ref					ref					ref					ref				
	Change*60-74	-0.019	0.019	0.331	-0.057	0.019	-0.461	0.228	**0.043**	-0.909	-0.014	-0.082	0.084	0.326	-0.246	0.082	-0.014	0.016	0.408	-0.046	0.019
	Change*75 and over	-0.050	0.020	**0.013**	-0.090	-0.010	-0.244	0.251	0.332	-0.736	0.249	-0.012	0.093	0.895	-0.195	0.170	-0.017	0.017	0.328	-0.051	0.017
Sex	Change*Men	ref					ref					ref					ref				
	Change*Women	0.032	0.015	**0.036**	0.002	0.061	0.996	0.179	**0.000**	0.646	1.347	0.228	0.064	**0.000**	0.102	0.354	0.020	0.012	0.110	-0.005	0.044
Wealth (tertiles)	Change*1st tertile	0.003	0.019	0.858	-0.033	0.040	-0.687	0.212	**0.001**	-1.102	-0.272	-0.191	0.077	**0.013**	-0.341	-0.041	-0.019	0.015	0.190	-0.048	0.010
	Change*2nd tertile	0.003	0.016	0.836	-0.028	0.035	-0.318	0.195	0.104	-0.700	0.065	-0.123	0.067	0.066	-0.255	0.008	0.001	0.012	0.904	-0.023	0.026
	Change*3rd tertile	ref					ref					ref					ref				
Partnership	Change*Partnered	ref					ref					ref					ref				
	Change*Non-partnered	0.021	0.019	0.273	-0.016	0.058	-0.066	0.216	0.758	-0.490	0.357	0.171	0.080	**0.032**	0.014	0.327	-0.013	0.014	0.342	-0.041	0.014

Note. *ELSA COVID-19 longitudinal sample (N=4867); weighted pooled estimates across 20 imputed datasets; p-values highlighted in bold are statistically significant at the 95% confidence level; interaction effects of sociodemographic characteristics with change before and during COVID-19 (average).*

© 2021 Zaninotto P et al. *JAMA Psychiatry*.

eTable 12. Two-way fixed-effects models: changes in mental health before and during the COVID-19 pandemic (robust standard errors)

	Estimate (b)	SE	p-value	CI (lower)	CI (upper)
**Outcome: Depression**					
Change before vs during COVID-19 (average)	0.131	0.007	**0.000**	0.116	0.145
Change before vs during COVID-19 (w1)	0.101	0.008	**0.000**	0.085	0.117
Change before vs during COVID-19 (w2)	0.160	0.008	**0.000**	0.144	0.177
Change COVID-19 w1 vs w2	0.060	0.008	**0.000**	0.044	0.075
**Outcome: Poor Quality of Life**					
Change before vs during COVID-19 (average)	1.185	0.089	**0.000**	1.011	1.360
Change before vs during COVID-19 (w1)	0.919	0.099	**0.000**	0.725	1.112
Change before vs during COVID-19 (w2)	1.452	0.096	**0.000**	1.264	1.640
Change COVID-19 w1 vs w2	0.534	0.078	**0.000**	0.381	0.686
**Outcome: Loneliness**					
Change before vs during COVID-19 (average)	0.201	0.033	**0.000**	0.137	0.265
Change before vs during COVID-19 (w1)	0.151	0.036	**0.000**	0.081	0.221
Change before vs during COVID-19 (w2)	0.251	0.036	**0.000**	0.181	0.321
Change COVID-19 w1 vs w2	0.100	0.029	**0.001**	0.043	0.157
**Outcome: Anxiety**					
Change COVID-19 w1 vs w2	0.016	0.006	**0.008**	0.004	0.027
Note. *ELSA COVID-19 longitudinal sample (N=5146); weighted pooled estimates across 20 imputed datasets; p-values highlighted in bold are statistically significant at the 95% confidence level.*					

© 2021 Zaninotto P et al. *JAMA Psychiatry*.

eTable 13. Descriptive statistics of the mental health outcomes from wave 4 to COVID-19 wave 2

	Imputed data, weighted							
	**Wave 4 (2008/09)**	**Wave 5 (2010/11)**	**Wave 6 (2012/13)**	**Wave 7 (2014/15)**	**Wave 8 (2016/17)**	**Wave 9 (2018/19)**	**COVID-19 Wave 1 (June-July 2020)**	**COVID-19 Wave 2 (Nov-Dec 2020)**
**Elevated depressive symptoms (CESD-8 > 3)**								
No	83.70%	82.00%	84.10%	83.00%	83.90%	87.40%	77.40%	71.50%
Yes	16.30%	18.00%	15.90%	17.00%	16.10%	12.60%	22.60%	28.50%
**Poor Quality of Life (CASP-12)**								
Mean (SD)	23.453 (6.496)	22.790 (6.112)	23.273 (6.429)	22.174 (6.200)	21.802 (6.078)	21.619 (6.289)	22.529 (6.526)	23.062 (6.670)
Range	5.000 - 45.000	1.000 - 46.000	2.000 - 44.000	1.000 - 44.000	1.000 - 48.000	1.000 - 48.000	12.000 - 47.000	9.000 - 48.000
**Loneliness**								
Mean (SD)	6.114 (2.322)	5.730 (2.117)	5.857 (2.170)	5.654 (2.098)	5.748 (2.120)	5.493 (2.020)	5.648 (2.065)	5.748 (2.172)
Range	1.000 - 12.000	1.000 - 12.000	1.000 - 12.000	1.000 - 12.000	1.000 - 12.000	1.000 - 12.000	2.000 - 12.000	3.000 - 12.000
Note. *ELSA COVID-19 longitudinal sample (N=5146); SD = standard deviation.*								

© 2021 Zaninotto P et al. *JAMA Psychiatry*.

eTable 14. Two-way fixed-effects models: changes in mental health before (wave 4 to 9) and during the COVID-19 pandemic

	Estimate (b)	SE	p-value	CI (lower)	CI (upper)
**Outcome: Depression**					
Model 1: Wave 9 vs COVID-19 waves and earlier waves					
Wave 4	0.037	0.026	0.164	-0.015	0.088
Wave 5	0.054	0.027	**0.043**	0.002	0.107
Wave 6	0.033	0.023	0.140	-0.011	0.078
Wave 7	0.044	0.028	0.113	-0.010	0.098
Wave 8	0.035	0.017	**0.032**	0.003	0.068
Wave 9	ref				
COVID-19 wave 1	0.100	0.009	**0.000**	0.083	0.116
COVID-19 wave 2	0.159	0.008	**0.000**	0.143	0.176
Model 2: Pre-COVID-19 waves (average waves 4-9) vs COVID-19 waves					
Before COVID-19 (waves 4-9)	ref				
COVID-19 wave 1	0.066	0.014	**0.000**	0.038	0.094
COVID-19 wave 2	0.125	0.015	**0.000**	0.096	0.154
**Outcome: Poor Quality of Life**					
Model 1: Wave 9 vs COVID-19 waves and earlier waves					
Wave 4	1.834	0.538	**0.001**	0.779	2.888
Wave 5	1.171	0.392	**0.003**	0.403	1.939
Wave 6	1.654	0.431	**0.000**	0.810	2.498
Wave 7	0.555	0.276	**0.044**	0.014	1.097
Wave 8	0.183	0.429	0.670	-0.658	1.023
Wave 9	ref				
COVID-19 wave 1	0.910	0.102	**0.000**	0.709	1.110
COVID-19 wave 2	1.443	0.099	**0.000**	1.249	1.638
Model 2: Pre-COVID-19 waves (average waves 4-9) vs COVID-19 waves					
Before COVID-19 (waves 4-9)	ref				
COVID-19 wave 1	0.010	0.291	0.972	-0.560	0.580
COVID-19 wave 2	0.544	0.291	0.062	-0.026	1.114
**Outcome: Loneliness**					
Model 1: Wave 9 vs COVID-19 waves and earlier waves					
Wave 4	0.621	0.169	**0.000**	0.290	0.951
Wave 5	0.236	0.125	0.059	-0.009	0.482
Wave 6	0.363	0.117	**0.002**	0.133	0.593
Wave 7	0.160	0.141	0.254	-0.115	0.436
Wave 8	0.254	0.133	0.056	-0.007	0.515
Wave 9	ref				
COVID-19 wave 1	0.155	0.035	**0.000**	0.086	0.223
COVID-19 wave 2	0.255	0.035	**0.000**	0.186	0.324
Model 2: Pre-COVID-19 waves (average waves 4-9) vs COVID-19 waves					
Before COVID-19 (waves 4-9)	ref				
COVID-19 wave 1	-0.118	0.090	0.189	-0.293	0.058
COVID-19 wave 2	-0.018	0.090	0.844	-0.195	0.159

© 2021 Zaninotto P et al. *JAMA Psychiatry*.

Note. *ELSA COVID-19 longitudinal sample (N=5146); weighted pooled estimates across 20 imputed datasets; p-values highlighted in bold are statistically significant at the 95% confidence level.*

© 2021 Zaninotto P et al. *JAMA Psychiatry*.

eFigure 3. Predicted trajectories of depression, poor quality of life, and loneliness before (waves 4 to 9) and during the COVID-19 pandemic (fixed-effects models)

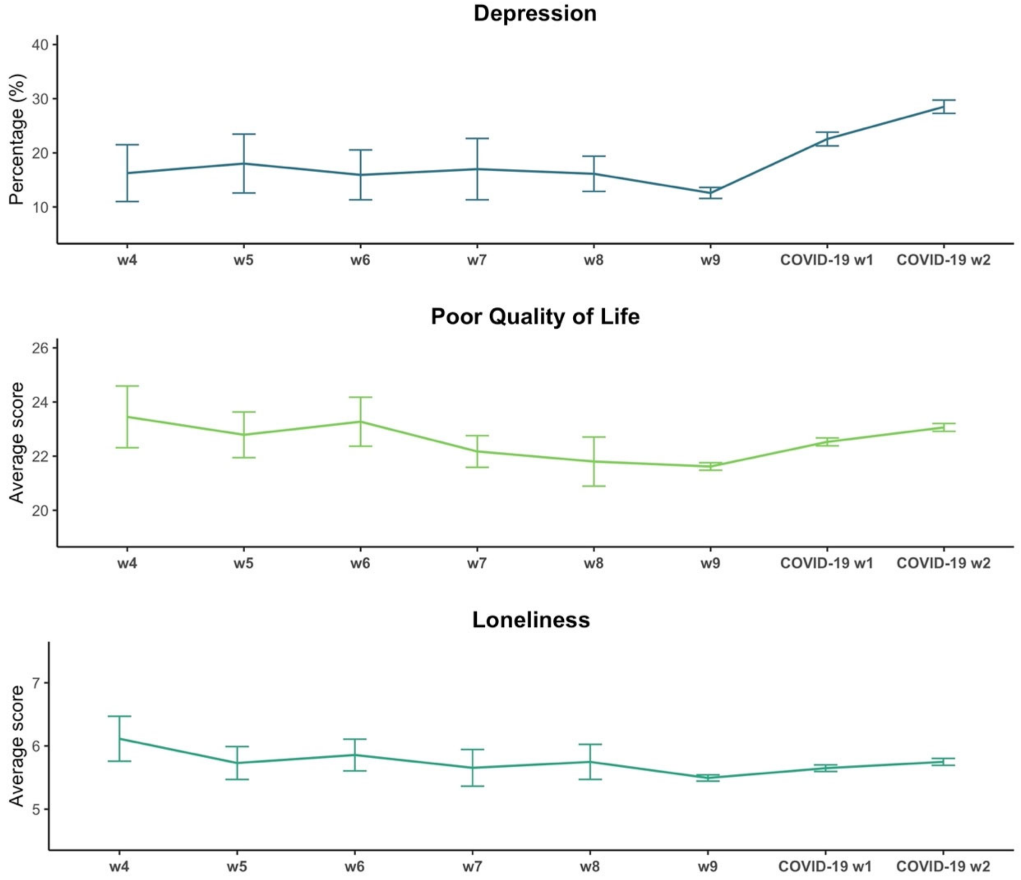

Note. *ELSA COVID-19 longitudinal sample (N=5,146); weighted pooled estimates from two-way fixed-effects linear models across 20 imputed datasets.*

© 2021 Zaninotto P et al. *JAMA Psychiatry*.

eTable 15. Comparison of the differences in loneliness and poor quality of life scores between people with and without chronic physical illnesses at wave 9 vs the changes in loneliness and poor quality of life scores before (wave 9) and during the COVID-19 pandemic (wave 2)

**Outcome**	**Exposure**	**B**	**SE**	**p-value**	**CI (lower)**	**CI (upper)**
Loneliness	Cancer (w9)	0.184	0.087	**0.035**	0.013	0.356
	Cardiovascular disease (w9)	0.266	0.098	**0.007**	0.074	0.458
	Change before (w9) vs during COVID-19 (w2)	0.252	0.035	**0.000**	0.183	0.322
Poor Quality of Life	Cancer (w9)	0.975	0.271	**0.000**	0.443	1.506
	Cardiovascular disease (w9)	2.206	0.303	**0.000**	1.613	2.800
	Change before (w9) vs during COVID-19 (w2)	1.461	0.096	**0.000**	1.272	1.649

© 2021 Zaninotto P et al. *JAMA Psychiatry*.

eReferences.

1 Radloff LS. The CES-D Scale: A Self-Report Depression Scale for Research in the General Population. *Appl Psychol Meas* 1977; **1**: 385–401.

2 Steffick D. Documentation of Affective Functioning Measures in the Health and Retirement Study. Ann Arbor, Michigan, 2000. https://hrs.isr.umich.edu/publications/biblio/5411 (accessed 8 Jul2020).

3 Wiggins RD, Netuveli G, Hyde M, Higgs P, Blane D. The Evaluation of a Self-enumerated Scale of Quality of Life (CASP-19) in the Context of Research on Ageing: A Combination of Exploratory and Confirmatory Approaches. *Soc Indic Res* 2007; **89**: 61–77.

4 Hughes ME, Waite LJ, Hawkley LC, Cacioppo JT. A Short Scale for Measuring Loneliness in Large Surveys: Results From Two Population-Based Studies. *Res Aging* 2004; **26**: 655–672.

5 Spitzer RL, Kroenke K, Williams JBW, Löwe B. A brief measure for assessing generalized anxiety disorder: The GAD-7. *Arch Intern Med* 2006; **166**: 1092–1097.

6 Eekhout I, de Vet HCW, Twisk JWR, Brand JPL, de Boer MR, Heymans MW. Missing data in a multi-item instrument were best handled by multiple imputation at the item score level. *J Clin Epidemiol* 2014; **67**: 335–342.

7 Rubin DB. *Multiple Imputation for Nonresponse in Surveys*. John Wiley and Sons: New York, 2004.

© 2021 Zaninotto P et al. *JAMA Psychiatry*.
